# Aspergillus niger, an Unusual Case With Fatal Massive Hemoptysis: A Case Report

**DOI:** 10.7759/cureus.106993

**Published:** 2026-04-13

**Authors:** Camila Baas-Yama, Xenia Lizeth Choc-Cano, Ivan Zepeda Quiroz, Maricarmen Pelayo-Madrigal, Daniel Juarez

**Affiliations:** 1 Internal Medicine, Hospital General de Zona 18, Instituto Mexicano del Seguro Social, Playa del Carmen, MEX; 2 Internal Medicine, Hospital de Traumatología y Ortopedia Dr. y General Rafael Moreno Valle, Puebla, MEX

**Keywords:** hemoptisis, pulmonary aspergillosis, pulmonary cavitation, rare case report, subacute pulmonary aspergillosis

## Abstract

*Aspergillus Niger *is considered a low-virulence fungus. Primary and secondary immunocompromise represent the principal risk factors for developing this disease. There are only a few cases of invasive infection that have been documented, and only a few fatal cases of secondary invasive pulmonary aspergillosis as a cavitation by this agent have been published. We present the case of a 45-year-old man with immunocompromise who developed massive hemoptysis, which, despite treatment, led to his death. Pathological findings documented invasive pulmonary aspergillosis due to *A. Niger* and a co-infection with multi-drug-resistant *Pseudomonas aeruginosa*. Invasive pulmonary aspergillosis is a fatal infection often accompanied by bacterial co-infection. This case demonstrates the potentially aggressive nature of *A. niger*, which may lead to high mortality hemoptysis. Timely treatment of cavitations through resection of lesions may prevent complications, as well as the early initiation of treatment. This case emphasizes that *A. niger*, although considered a low-virulence organism, can lead to aggressive and life-threatening disease, highlighting the need for early diagnosis and timely intervention to prevent fatal outcomes.

## Introduction

Pulmonary aspergillosis is a fungal infection with mortality rates ranging from 50% to 80%, caused by *Aspergillus* species, most commonly *Aspergillus fumigatus*, *Aspergillus flavus*, *Aspergillus terreus*, and *Aspergillus niger* [1,2]. It is estimated that invasive aspergillosis affects over two million individuals annually, primarily among immunocompromised patients, while chronic pulmonary aspergillosis affects approximately 1.8 million people worldwide each year [2].

*Aspergillus fumigatus* is the main causative agent of human infections due to its ability to adhere to the respiratory epithelium, its high thermotolerance, and its capacity to evade the host immune response, including reduced susceptibility to phagocytosis and modulation of the complement system, in contrast to other species such as *A. niger*, which exhibits lower virulence [3]. Pulmonary aspergillosis encompasses a wide clinical spectrum ranging from allergic and chronic forms to saprophytic colonization and invasive disease, depending on the host’s immune status, with invasive aspergillosis representing the most severe and life-threatening presentation [4].

There are limited case reports of *A. niger*, and 4% of them were published in only one cohort study. Primary and secondary immunocompromise represent the principal risk factor for developing this disease, for example, the reduced activity of phagocytes in diabetes [1-6]. Furthermore, aspergillosis may occur with bacterial co-infections, which increase mortality; Cho et al. reported a prevalence of 21.8% of bacterial co-infection with *Aspergillus* in Korea [7].

*Aspergillus niger* is generally considered a low-virulence fungus, not frequently reported as a cause of invasive infections. Only a few fatal cases of secondary invasive pulmonary aspergillosis by this agent have been published, and we have not found other cases with a cavitary lesion with the same characteristics seen in our patient. The objective of this case report is to show the aggressive presentation of a fungus considered to be of low virulence and the unusual radiological findings of a cavitation that can show this disease and generate massive hemoptysis, as well as evidence of co-infection with multidrug-resistant *Pseudomonas aeruginosa*.

## Case presentation

The patient is a 45-year-old male with a two-year history of poorly controlled diabetes mellitus and poor adherence to follow-up. He was being treated with metformin alone. Three weeks before admission, he presented with a fever of up to 39.0 °C, a productive cough, and moderate exertional dyspnea; he subsequently developed dysphonia and pleuritic pain. A non-contrast chest CT scan was subsequently performed, demonstrating atelectasis, cavitation, consolidation, and diffuse destruction of the pulmonary parenchyma in the right lung, predominantly involving the middle and lower lobes. A well-defined myzcetoma was clearly identified. A sputum culture was performed, revealing the presence of A. niger, and treatment with itraconazole was initiated in the hospital setting due to the lack of other antifungal agents. 

**Figure 1:**
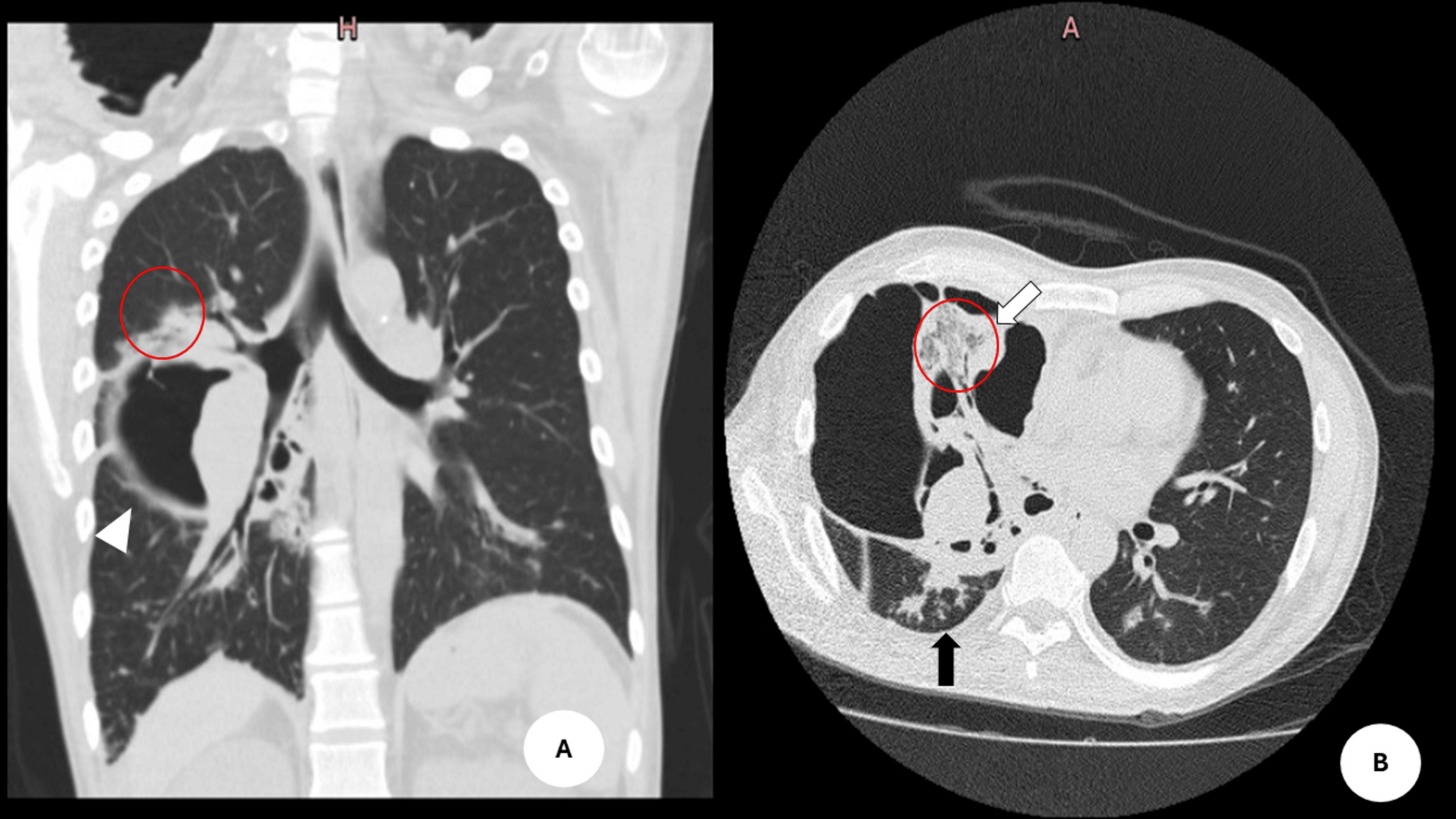
Pulmonary CT scans A: Axial section showing an extensive cavitary lesion in the right middle lobe with thickened walls (white arrowhead). The cavity contains solid soft-tissue components (black arrowhead), with associated findings including an air bronchogram (red circle), bronchial wall thickening, and a tree-in-bud pattern (black arrow), consistent with endobronchial involvement. B: Coronal reconstruction showing the same cavitary lesion, with better visualization of its extent and involvement of the upper pulmonary segments (red circle), as well as bronchial involvement (dotted arrow).

On admission, his vital signs were within normal limits, and laboratory tests showed no significant abnormalities; these values correspond to his admission to our hospital unit (Table 1). It is noteworthy that his initial hemoglobin level prior to transfer was 14.4 g/dL. At our hospital, a non-contrast chest CT scan revealed pulmonary cavitations in the right upper and middle lobes, as well as a right parapneumonic pleural effusion (Figure 1).

**Table 1:** Laboratory test values during hospitalization

Laboratory test	At the time of admission	At discharge and prior to death	Reference values
Leucocytes (10*3/uL)	10.52	7.84	4.5-10 (10*3/uL)
Hemoglobin (g/dL)	8.50	7.8	12-16 g/dL
Hematocrit (%)	25.2 %	23.3 %	36%-48%
Mean corpuscular volumen (fL)	82.6	79.0	80-100 fL
Mean corpuscular hemoglobin (pg)	27.9	26.4	28-32 pg
Mean corpuscular hemoglobin concentration (g/dL)	33.7	33.5	31-36 g/dL
Platelets (10*3/uL)	353.0	283.0	150-400 (10*3/uL)
Neutrophils (segmented) (10*3/uL)	7.13	5.32	1.5-8 (10*3/uL)
Lymphocytes (10*3/uL)	2.00	1.55	1-4.8 (10*3/uL)
Monocytes (10*3/uL)	1.22	0.88	0.1-0.9 (10*3/uL)
Eosinophils (10*3/uL)	0.12	0.08	0-0.5 (10*3/uL)
Basophils (10*3/uL)	0.05	0.01	0-0.2 (10*3/uL)
Glucose (mg/dL)	127.72	89.8	70-100 mg/dL
Blood urea nitrogen (mg/dL)	23.2	27.7	7-20 mg/dL
Serum creatinine (mg/dL)	1.09	0.73	0.6-1.2 mg/dL
Lactate dehydrogenase (U/L)	172	180	135-225 U/L
Alanine aminotransferase (U/L)	8	16.7	<42 U/L
Aspartate aminotransferase (U/L)	14	13.4	<40 U/L
Total bilirubin (mg/dL)	0.36	0.32	<2 mg/dL
C-reactive protein (mg/L)	6.0	3.5	<5 mg/L

Additionally, based on the radiological findings, three sputum smears and cultures were obtained to rule out tuberculosis. Advanced diagnostic tests such as galactomannan, beta-D-glucan, specific PCR, and bronchoscopy were not performed due to resource limitations at our hospital. Subsequently, evaluation by the thoracic surgery team was requested in light of these findings. Further assessment was also conducted to rule out other conditions associated with immunosuppression. 

**Figure 2:**
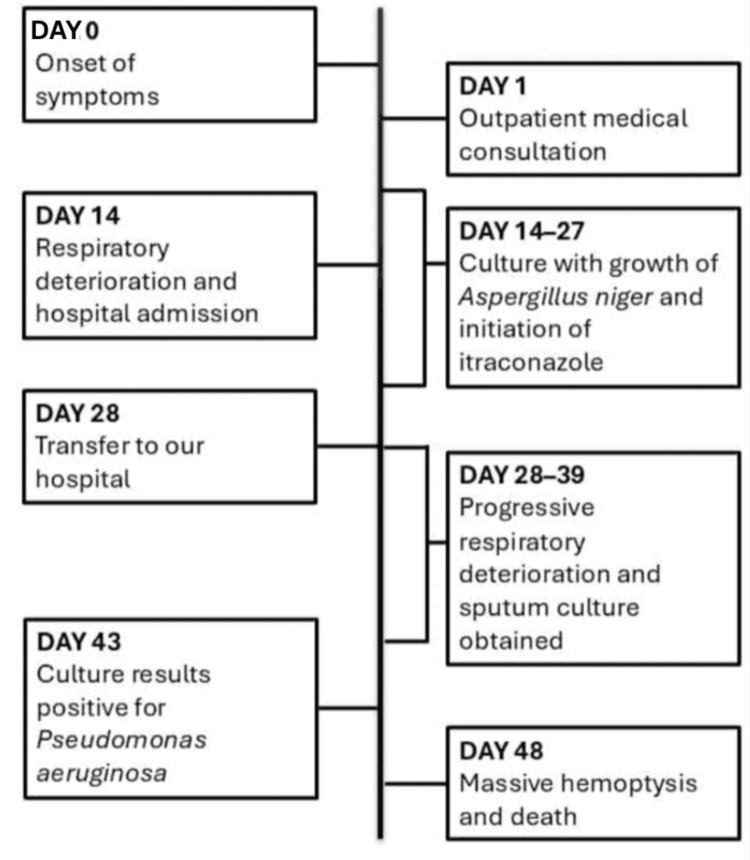
Timeline of clinical course and disease progression

The initial response to itraconazole was adequate, with no fever during the first seven days. However, while awaiting evaluation for thoracic surgery, which was not available at the facility, the patient developed a fever again, prompting a new sputum culture, from which *P. aeruginosa* was isolated. Nebulized amikacin and colistin were added to the treatment regimen. Itraconazole was administered at the maximum recommended dose for 22 days. Two days after starting colistin, the patient developed massive hemoptysis, leading to cardiorespiratory arrest that was refractory to resuscitation efforts. Due to the sudden and unexpected deterioration, laboratory tests were not performed to assess post-event hemoglobin levels (Figure 2).

## Discussion

Pulmonary aspergillosis has a wide range of clinical presentations, including invasive pulmonary aspergillosis (IPA), chronic necrotizing aspergillosis (sub-acute invasive pulmonary aspergillosis), aspergilloma, chronic cavitary pulmonary aspergillosis (CCPA), and allergic bronchopulmonary aspergillosis. The clinical presentation may depend on the patient’s immune status [1-6]. 

Currently, *Aspergillus* infections cause high morbidity and mortality. *Aspergillus niger* is the least common cause of invasive disease; this is attributed to its low virulence, mainly due to physiological characteristics such as the size of* A. niger’*s conidia and spore-bridging structures, which limit its entry into the lower respiratory tract [7]. Additionally, its optimal growth temperature (30°C) hinders germination at human body temperature, and its acidophilic nature further restricts its development [8]. All these features explain why invasive infection by *A. niger* typically occurs only in the context of severe immunosuppression; however, reports of its pathogenicity are increasing. High clinical and radiological suspicion is crucial for early diagnosis and treatment [1-7]. From the clinical perspective, IPA symptoms may include cough, fever, chest or pleuritic pain, dyspnea, and/or hemoptysis, which is one of the most severe complications of the disease. This presentation occurs in 64% to 83% of cases with aspergilloma, is rarely associated with *A. niger*, and carries high mortality [5]. However, patients with significant immunosuppression may not present classic symptoms due to the lack of an inflammatory response, which results in delayed diagnosis and a high rate of complications [8-12].

The diagnosis of IPA should be established based on the European Organization for Research and Treatment of Cancer/Invasive Fungal Infections Cooperative Group and the National Institute of Allergy and Infectious Diseases Mycoses Study Group (EORTC/MSG) criteria, which are based on the host (immunosuppressive conditions), clinical (clinical and radiological signs), and mycological criteria. The aim is to classify them on a probability scale: possible (at least one host criterion and one clinical criterion), probable (one host criterion, one clinical criterion, and one mycological criterion), and proven (regardless of the presence or absence of host/clinical/mycological criteria) [12]. A CT scan should always be performed when IPA is suspected in an immunocompromised patient. Other studies include histological examination of biopsy or surgical samples and culture methods and non-culture methods, such as fungal biomarkers (galactomannan, 1,3-β-d-glucan (BDG)) and PCR [13,14]. 

Treatment includes voriconazole as the primary drug and has shown better survival rates when compared to amphotericin B or isavuconazole sulfate. It is important to mention that voriconazole has non-linear pharmacokinetics, leading to variability in serum levels in healthy hosts. The main explanation for the wide variation in serum levels is the ability of hosts to metabolize voriconazole through the CYP450 enzyme, which generates controversy regarding the need for monitoring in patients with similar characteristics to our patient [15]. In cases of resistance to azoles, voriconazole and echinocandins or liposomal amphotericin B are recommended for six to 12 weeks, depending on severity and treatment response [16,17].

The hemoptysis starts with chronic or repeated episodes of infection and inflammation, leading to dilation and distortion of the bronchial arteries. These inflammatory processes enhance the normal vascular connections between bronchial and pulmonary vessels, resulting in increased blood flow through the now-enlarged bronchial arteries. Moreover, the release of angiogenic factors, such as vascular endothelial growth factor, stimulates the formation of fragile new and collateral vessels, which possess thin walls and are susceptible to rupture [18]. The management of massive hemoptysis requires immediate intervention focused on securing the airway, hemodynamic stabilization, and identifying the source of bleeding. It is essential to position the patient in lateral decubitus with the bleeding lung dependent, administer supplemental oxygen, and consider orotracheal intubation with a large-caliber tube or selective intubation, depending on the case, to protect the unaffected lung. Bronchoscopy, whether flexible or rigid, is useful for both diagnosis and initial treatment, allowing for localization of the bleeding site and the application of measures such as instillation of hemostatic agents, topical vasoconstrictors, coagulation, or tamponade. If bleeding persists, bronchial artery embolization is the most effective and least invasive non-surgical therapeutic option, with a high success rate and low risk of complications. In refractory cases, surgical pulmonary resection may be necessary, although it carries a higher risk and should be reserved for carefully selected patients [19,20]. 

In this patient, the clinical features reflect the dynamic nature of *Aspergillus* infection; cough, pleuritic chest pain, dyspnea, and hemoptysis were present. Laboratory findings were consistent with vascular involvement. However, sudden deterioration associated with massive hemoptysis is more commonly observed in aspergilloma, where *A. fumigatus* is the usual causative agent. In contrast, the organism isolated in this case was *A. niger*. Notably, unlike most previously reported cases, this patient had no history of immunosuppression or underlying pulmonary disease described in the literature. This case provides additional clinical insight into the behavior of *A. niger*, suggesting that although it is generally considered a low-virulence organism, it may exhibit an aggressive course in patients with poor metabolic control and bacterial co-infection, in this case, *P. aeruginosa*. Furthermore, it highlights how the absence of specialized interventions may directly contribute to an unfavorable prognosis.

## Conclusions

We presented the case of a young patient with poor metabolic control and an infection caused by *A. niger*, which is a less common agent compared to *A. fumigatus* and other *Aspergillus* species, with a rare and fatal presentation. Although *A. niger* is considered a low-virulence pathogen, it can cause severe pulmonary disease in patients with risk factors. It also underscores the importance of timely diagnostic evaluation and a multidisciplinary approach in the presence of cavitary lung lesions, as delays in management may lead to potentially fatal complications such as massive hemoptysis.
